# Journal of Insanity

**Published:** 1869-05

**Authors:** 


Journal of Insanity.—The American Journal of Insanity comes to us, containing in addition to its usual contents of interesting original matter, stereoscopic representations of the brain in a case of apoplexy, and also a very fine photograph of Prof. William Griesinger, to accompany a memoir of his life. The number contains other representations of disease, and is one of which the editors and publishers may rightfully feel proud.

